# The correlation between microvessel pathological changes of the endplate and degeneration of the intervertebral disc in diabetic rats

**DOI:** 10.3892/etm.2020.9129

**Published:** 2020-08-21

**Authors:** Sen Chen, Meimei Liao, Jianping Li, Hao Peng, Min Xiong

Exp Ther Med 5:711–717, 2013; DOI: 10.3892/etm.2012.868

Following the publication of the above paper, an interested reader drew to our attention apparent anomalies associated with Figs. 3 and 4. Essentially, overlapping data featured in 6 of the 12 panels in Figs. 3A-F and 4A-F appeared to have been derived from no more than two original images. Therefore, several data panels purportedly relating to different experiments were apparently drawn from the same original sources.

After having investigated this matter, the Editor of *Experimental and Therapeutic Medicine* has upheld the claims made by the interested reader, and a decision has been taken that this paper should be retracted from the Journal. All the authors agree to this retraction; furthermore, the authors regret any inconvenience caused to the readership of the Journal.

